# Supplementary material for: Optimizing NPSB fertilizer rates for enhanced yield and yield components of mung bean (Vigna radiata L:) varieties
Source: PLoS One. 2025 Oct 24;20(10):e0321243. doi: 10.1371/journal.pone.0321243 (PMC12551827; doi:10.1371/journal.pone.0321243)
Supplement: Attachment — Submitted filename: PONE-D-25-10453.pdf [file pone.0321243.s001.pdf]

PLOS ONE

Optimizing NPSB Fertilizer Application Rates for Enhanced Yield and Yield Components of Mung Bean (Vigna Radiata L.) Varieties in the Dalocha District of Central Ethiopia

--Manuscript Draft--

**Manuscript Number:**	PONE-D-25-10453
**Article Type:**	Research Article
**Full Title:**	Optimizing NPSB Fertilizer Application Rates for Enhanced Yield and Yield Components of Mung Bean (Vigna Radiata L.) Varieties in the Dalocha District of Central Ethiopia
**Short Title:**	Optimizing NPSB Fertilizer Application Rates for Yield and Yield Components of Mung Bean in Ethiopia
**Corresponding Author:**	Daniel Manore<br>Wachemo University<br>Hossana, ETHIOPIA
**Keywords:**	Ethiopia; Net benefit; Rates; Soil fertility; Varietal performance
**Abstract:**	Mung bean cultivation in Ethiopia faces several challenges, including low soil fertility, limited application of NPSB fertilizers, and restricted access to improved varieties. This study evaluated the effects of different mung bean varieties and NPSB fertilizer rates on crop yield. A field experiment was conducted over a two-year period (2022 and 2023) using a factorial design with three mung bean varieties (NVL-1, N26, and Arkebe) and five NPSB fertilizer rates (0, 25, 50, 75, and 100 kg/ha). The treatments were arranged in a randomized complete block design with three replications. Soil properties were analyzed before and after the experiment using standard laboratory methods. The results revealed that NPSB fertilizer significantly influenced soil pH, available boron, total nitrogen, and organic carbon ($p < 0.05$), while cation exchange capacity, phosphorus, and sulfur remained unaffected. The interaction between mung bean varieties and fertilizer rates significantly impacted soil pH, cation exchange capacity, and organic carbon. Among the treatments, the N26 variety combined with 100 kg/ha NPSB fertilizer produced the highest number of pods per plant, seeds per pod, fresh and dry leaf weight, leaf area, harvest index, grain yield, and biomass in both years. In 2023, N26 achieved the highest grain yield of 1.94 tonnes/ha, while Arkebe without fertilizer recorded the lowest yield at 1.27 tones/ha. Additionally, N26 generated the highest net benefit, amounting to 47,704.17 ETB/ha in 2022 and 49,856.85 ETB/ha in 2023. The study recommends the application of 100 kg/ha NPSB fertilizer with the N26 variety to maximize productivity. Further research is needed to validate these findings under diverse environmental conditions.
**Order of Authors:**	Daniel Manore
	Hiwot Kelbo
	Oumer Abdella
	Dr Francis Abuye
**Additional Information:**	
**Question**	**Response**
**Financial Disclosure**<br><br>Enter a financial disclosure statement that describes the sources of funding for the work included in this submission. Review the submission guidelines for detailed requirements. View published research articles from *PLOS ONE* for specific	The author(s) received no specific funding for this work.

Powered by Editorial Manager® and ProduXion Manager® from Aries Systems Corporation

examples.<br><br>This statement is required for submission and **will appear in the published article** if the submission is accepted. Please make sure it is accurate.<br><br>**Funded studies**<br>Enter a statement with the following details:<br>• Initials of the authors who received each award<br>• Grant numbers awarded to each author<br>• The full name of each funder<br>• URL of each funder website<br>• Did the sponsors or funders play any role in the study design, data collection and analysis, decision to publish, or preparation of the manuscript?<br><br>Did you receive funding for this work?	
**Competing Interests**<br><br>Use the instructions below to enter a competing interest statement for this submission. On behalf of all authors, disclose any competing interests that could be perceived to bias this work—acknowledging all financial support and any other relevant financial or non-financial competing interests.<br><br>This statement is required for submission and **will appear in the published article** if the submission is accepted. Please make sure it is accurate and that any funding sources listed in your Funding Information later in the submission form are also declared in your Financial Disclosure statement.<br><br>View published research articles from *PLOS ONE* for specific examples.	The authors have declared that no competing interests exist.

**NO authors have competing interests**<br><br>Enter: *The authors have declared that no competing interests exist.*<br><br>**Authors with competing interests**<br><br>Enter competing interest details beginning with this statement:<br><br>*I have read the journal's policy and the authors of this manuscript have the following competing interests: [insert competing interests here]*<br><br>* typeset	
**Ethics Statement**<br><br>Enter an ethics statement for this submission. This statement is required if the study involved:<br><br>• Human participants<br>• Human specimens or tissue<br>• Vertebrate animals or cephalopods<br>• Vertebrate embryos or tissues<br>• Field research<br><br>Write "N/A" if the submission does not require an ethics statement.<br><br>General guidance is provided below. Consult the submission guidelines for detailed instructions. **Make sure that all information entered here is included in the Methods section of the manuscript.**	N/A

Powered by Editorial Manager® and ProduXion Manager® from Aries Systems Corporation

**Format for specific study types**<br><br>**Human Subject Research (involving human participants and/or tissue)**<br>• Give the name of the institutional review board or ethics committee that approved the study<br>• Include the approval number and/or a statement indicating approval of this research<br>• Indicate the form of consent obtained (written/oral) or the reason that consent was not obtained (e.g. the data were analyzed anonymously)<br><br>**Animal Research (involving vertebrate animals, embryos or tissues)**<br>• Provide the name of the Institutional Animal Care and Use Committee (IACUC) or other relevant ethics board that reviewed the study protocol, and indicate whether they approved this research or granted a formal waiver of ethical approval<br>• Include an approval number if one was obtained<br>• If the study involved *non-human primates*, add *additional details* about animal welfare and steps taken to ameliorate suffering<br>• If anesthesia, euthanasia, or any kind of animal sacrifice is part of the study, include briefly which substances and/or methods were applied<br><br>**Field Research**<br><br>Include the following details if this study involves the collection of plant, animal, or other materials from a natural setting:<br>• Field permit number<br>• Name of the institution or relevant body that granted permission	
**Data Availability**<br><br>Authors are required to make all data underlying the findings described fully available, without restriction, and from the time of publication. PLOS allows rare exceptions to address legal and ethical concerns. See the PLOS Data Policy and FAQ for detailed information.	Yes - all data are fully available without restriction

Powered by Editorial Manager® and ProduXion Manager® from Aries Systems Corporation

A Data Availability Statement describing where the data can be found is required at submission. Your answers to this question constitute the Data Availability Statement and **will be published in the article**, if accepted.<br><br>**Important:** Stating 'data available on request from the author' is not sufficient. If your data are only available upon request, select 'No' for the first question and explain your exceptional situation in the text box.<br><br>Do the authors confirm that all data underlying the findings described in their manuscript are fully available without restriction?	
**Describe where the data may be found in full sentences. If you are copying our sample text, replace any instances of XXX with the appropriate details.**<br><br>• If the data are **held or will be held in a public repository**, include URLs, accession numbers or DOIs. If this information will only be available after acceptance, indicate this by ticking the box below. For example: *All XXX files are available from the XXX database (accession number(s) XXX, XXX.*).<br>• If the data are all contained **within the manuscript and/or Supporting Information files**, enter the following: *All relevant data are within the manuscript and its Supporting Information files.*<br>• If neither of these applies but you are able to provide **details of access elsewhere**, with or without limitations, please do so. For example:<br><br>*Data cannot be shared publicly because of [XXX]. Data are available from the XXX Institutional Data Access / Ethics Committee (contact via XXX) for researchers who meet the criteria for access to confidential data.*<br><br>*The data underlying the results presented in the study are available from (include the name of the third party*	The data underlying the results presented in the study are available from corresponding author

and contact information or URL).<br>• This text is appropriate if the data are owned by a third party and authors do not have permission to share the data.<br><br>* typeset	
Additional data availability information:	Tick here if the URLs/accession numbers/DOIs will be available only after acceptance of the manuscript for publication so that we can ensure their inclusion before publication.

Manuscript

Click here to access/download;Manuscript;Mung bean paper @ Dani.docx

Optimizing NPSB Fertilizer Application Rates for Enhanced Yield and Yield Components of Mung Bean (*Vigna Radiata* L.) Varieties in the Dalocha District of Central Ethiopia

Daniel Manore1 daniel.manore@wcu.edu.et*, Hiwot Kelbo[1] hiwotk@wcu.edu.et, Oumer Abdella[2] email oumerabdella2011@gmail.com, Francis Abuye[3] fabuye649@gmail.com

Wachemo University Department of Plant science, Hossaena Ethiopia[1] P.O.Box 667

Silte Zone Agricultural office, Department of field Crops, Worabe Ethiopa[2] P.O.Box 08

Wachemo University Department of Natural Resource Management Hossaena Ethiopia[3] P.O.Box 667

Cross ponding author daniel.manore@wcu.edu.et*

Abstract

Mung bean cultivation in Ethiopia faces several challenges, including low soil fertility, limited application of NPSB fertilizers, and restricted access to improved varieties. This study evaluated the effects of different mung bean varieties and NPSB fertilizer rates on crop yield. A field experiment was conducted over a two-year period (2022 and 2023) using a factorial design with three mung bean varieties (NVL-1, N26, and Arkebe) and five NPSB fertilizer rates (0, 25, 50, 75, and 100 kg/ha). The treatments were arranged in a randomized complete block design with three replications. Soil properties were analyzed before and after the experiment using standard laboratory methods. The results revealed that NPSB fertilizer significantly influenced soil pH, available boron, total nitrogen, and organic carbon ($p < 0.05$), while cation exchange capacity, phosphorus, and sulfur remained unaffected. The interaction between mung bean varieties and fertilizer rates significantly impacted soil pH, cation exchange capacity, and organic carbon. Among the treatments, the N26 variety combined with 100 kg/ha NPSB fertilizer produced the highest number of pods per plant, seeds per pod, fresh and dry leaf weight, leaf area, harvest index, grain yield, and biomass in both years. In 2023, N26 achieved the highest grain yield of 1.94 tonnes/ha, while Arkebe without fertilizer recorded the lowest yield at 1.27 tones/ha. Additionally, N26 generated the highest net benefit, amounting to 47,704.17 ETB/ha in 2022 and 49,856.85 ETB/ha in 2023. The study recommends the application of 100 kg/ha NPSB fertilizer with the N26 variety to maximize productivity. Further research is needed to validate these findings under diverse environmental conditions.

Keywords: Ethiopia; Net benefit**;** Rates; Soil fertility; Varietal performance

Introductions

Mung bean (*Vigna radiata* L.), or green gram, is a vital pulse crop in Southeast Asia [1]. It is a member of the Papilionoideae family, it thrives in warm climates, requires minimal water, and enhances soil fertility through nitrogen fixation, fixing approximately 47 kg of nitrogen per hectare [2]. Valued for its versatility, mung bean is used for human consumption (as dry beans or sprouts), livestock forage, and green manure. It is a nutrient-dense crop, providing 24-26% protein, essential amino acids, vitamins, and minerals at a low cost [3]. Its nutritional profile includes 1-3% fats, 50.4% carbohydrates, 3.5-4.5% fiber, and significant amounts of vitamin A, iron, calcium, zinc, and vitamin C, making it a valuable dietary component [4]. Additionally, mung beans offer health benefits such as reducing cholesterol and managing diabetes. In Ethiopia, mung bean cultivation spans 49,123.52 hectares, producing 557,928.53 quintals annually, with an average yield of 1.136 tons per hectare [5]. However, productivity lags behind other countries due to low soil fertility and inadequate use of inorganic fertilizers, particularly nitrogen (N), phosphorus (P), sulfur (S), and boron (B) [6]. These nutrients are crucial for plant growth, yield, and quality, and their deficiency has hindered mung bean production. Addressing soil fertility through balanced nutrient management is essential for improving yields [7].

Research by [8] highlights promising mung bean genotypes that could boost production in Ethiopia. However, challenges such as limited farmer experience, insufficient adoption of improved technologies, and improper agronomic practices persist. Additionally, a lack of information on optimal NPSB fertilizer rates and suitable varieties further constrains productivity. Sustainable agronomic practices and improved fertilizer application are critical to enhancing mung bean cultivation, especially under changing climatic conditions [9].

As a legume, mung bean is environmentally sustainable, requiring minimal water and restoring soil fertility. Its high protein and iron content make it a nutritious food source, vital for addressing food security and nutritional needs [7]. This study aims to evaluate the effects of NPSB fertilizer rates and mung bean varieties on yield, identify the most effective variety, and assess the economic feasibility of fertilizer application over two cropping years. By addressing these factors, mung bean production in Ethiopia can be significantly improved.

Objectives

• To determine the optimal NPSB fertilizer application rates for enhancing the yield of *Vigna radiata* L. in the Dalocha District of the Siltie Zone, Central Ethiopia.
• To investigate the impact of NPSB fertilizer application rates on yield components of mung bean in the Dalocha District.
• To evaluate the economic feasibility of NPSB fertilizer application for mung bean production in the Dalocha District

MATERIALS AND METHOD

Description of Study Area

Mataya Dange is located in the Dalocha district of the Silte zone, positioned at coordinates 9.1450° N and 40.4897° E, with an elevation of 1,954.25 meters (6,411.58 feet) above sea level, as illustrated in Fig. 1. The region experiences a tropical wet and dry (savanna) climate. It is situated approximately 132 km southwest of Addis Ababa, nestled between the towns of Hosanna and Butejira.

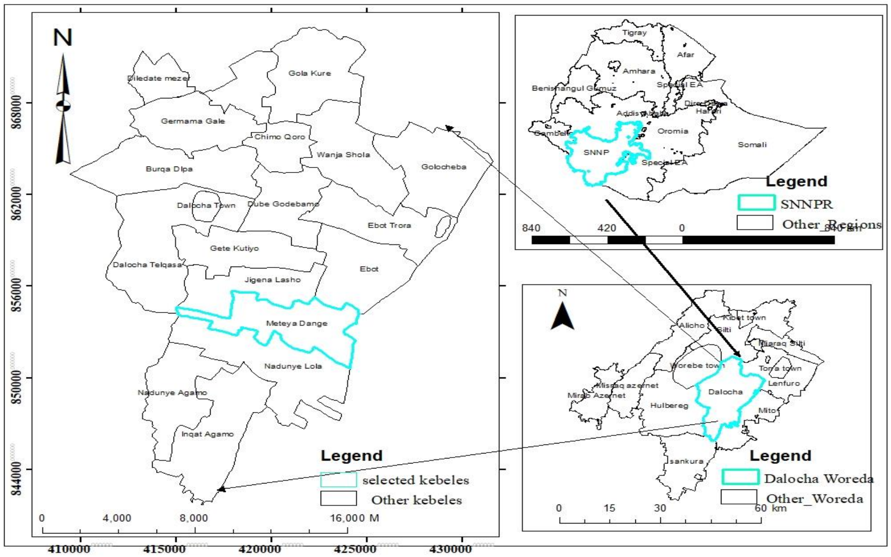

Figure 1:The geographical location of the experimental site.

Rain fall and temperature

The district's average annual temperature is 21.85°C, slightly below Ethiopia's national average, creating a mild climate suitable for agriculture. Dalocha receives about 161.3 mm (6.35 inches) of rainfall annually (Fig. 2), spread over approximately 200.64 rainy days, covering 54.97% of the year. This extended wet season supports diverse crop growth and sustains local ecosystems. Rainfall patterns are vital for residents' livelihoods, significantly impacting agricultural productivity and water availability.

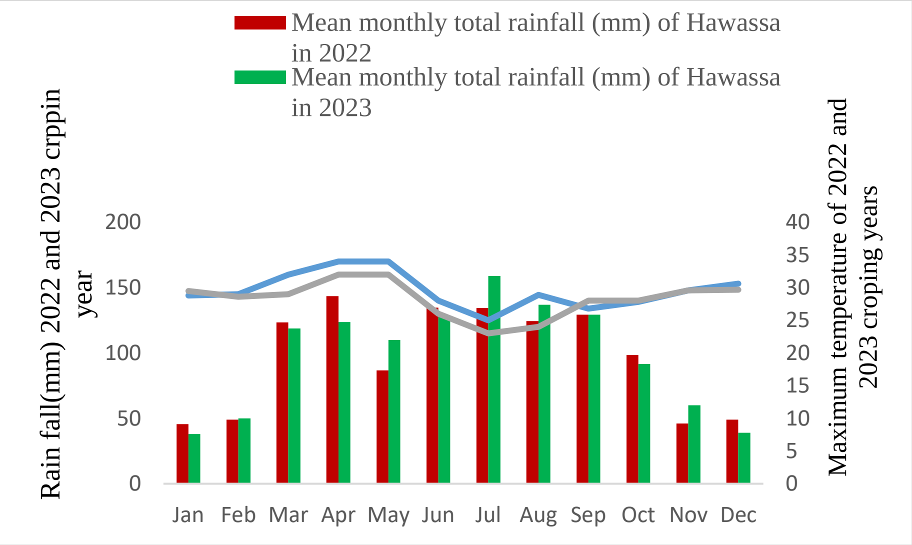

Figure 2:Weather data of cropping years' s at Dalocha

Experimental Materials Description

The blended NPSB fertilizer utilized in the experiment consisted of the following nutrient composition: 18.9% nitrogen, 37.75% P_2O_5, 6.95% sulfur, and 0.1% other elements. The selection of the blended NPSB fertilizer was based on soil fertility and fertilizer recommendations provided by atlas [10]. The mung bean varieties employed in this experiment were obtained from the Melkassa Agricultural Research Center. These varieties are known for their broad adaptability. A comprehensive description of the mung bean varieties used in the experiment is presented in Table

Table 1: Description of the experimental materials

Genotypes	Year of release	Seed size	Status	Source
N-26	2011	Small	Released variety	Melkasa ARC
Arkebe	2014	Small	Released variety	Humera ARC
NVL-1	2014	Small	Released variety	Melkasa ARC

ARC = Agricultural Research Center.

Treatments and Experimental Design

The experiment evaluated three Mung bean varieties—NVL-1, N-26, and Arkebe—combined with five NPSB fertilizer rates (0, 25, 50, 75, and 100 kg ha^{-1}), resulting in 15 treatments. Conducted over two cropping years (2021/22–2022/23), the study employed a factorial randomized complete block design (RCBD) with three replicates. Each $4m^2$ plot (2m × 2m) contained six rows, with 0.5m and 1m spacing between plots and blocks, respectively. The total experimental area was $296m^2$ (8m × 37m). Sowing was done at 30cm inter-row and 10cm intra-row spacing, with seeds planted at a 30cm depth.

Experimental Procedures

The field was plowed three times using oxen, leveled, and prepared. Sowing occurred on May 8, 2022, and 2023, during the medium rainfall season. Two seeds were planted per hole, and NPSB fertilizer was applied using the drill method at sowing, placed 5cm deep and covered with soil. Uniform agronomic practices, including weeding, spacing, and disease management, were maintained across all plots.

Soil Physicochemical Properties

Before the sowing process began, soil samples were collected from ten different locations at a depth of 0–30 cm using a zigzag sampling pattern. These individual samples were then mixed to form composite samples, ensuring a representative analysis of the soil. After the crops were harvested in both years of the study, soil samples were again collected from each treatment plot for further analysis.

The soil properties analyzed included texture, pH, organic carbon (OC), total nitrogen (TN), available phosphorus (AP), cation exchange capacity (CEC), available sulfur (AS), and available boron (AB). These analyses were conducted at Wachamo University using established methodologies. Specifically, soil pH was measured potentiometrically in a 1:2.5 soil-water suspension [11]. Organic carbon (OC) was determined using the Walkley-Black method [12], while total nitrogen (TN) was analyzed via the Kjeldahl method [13]. Available phosphorus (AP) was measured using the Olsen method [14], and available sulfur (AS) was analyzed turbidimetrically[15]. Soil texture was determined using the Bouyoucos hydrometer method [16],

and cation exchange capacity (CEC) was assessed using the ammonium acetate method [17]. Additionally, electrical conductivity (EC) was measured with an EC meter [17]. These comprehensive analyses were carried out both before planting and after harvest to monitor and evaluate changes in soil properties over the course of the study. This approach provided valuable insights into the impact of agricultural practices on soil health and fertility.

Growth and Phenological Data Collection

Phenological Data

Days of Emergence: The period from sowing until 50% of plants showed panicle tip emergence was recorded through visual observation.

Days of Physiological Maturity: The stage when 90% of plants in each plot reached physiological maturity was documented.

Nodule Number per Plant: At mid-flowering, five plants from the second inner rows were uprooted, washed gently, and separated into roots and shoots. Nodules were removed, spread on a sieve, and counted to determine the average nodules per plant.

2.6. Plant Measurements and Yield Assessment

Plant Height (cm): The average height of five randomly selected plants (excluding roots) was measured at harvest using a meter scale.

Number of Branches: Branches were counted at 90% maturity.

Leaf Area: Measured using a leaf area meter on five plants during peak vegetative growth.

Leaf Area Index (LAI): Calculated as the total leaf area of five plants (cm^2) divided by the land area they occupied.

Fresh and Dry Weight: Plants were patted dry, weighed for fresh weight, oven-dried, cooled in a desiccator, and reweighed for dry weight.

Number of Pods per Plant: Pods from five plants were counted to determine the average per plant.

Number of Seeds per Pod: Seeds in ten randomly selected pods per treatment were counted for the mean.

Thousand-Seed Weight (g): A 1,000-grain sample from each plot was weighed, adjusted to 11% moisture content.

Aboveground Biomass (kg/ha): Dry biomass yield was recorded by harvesting three central rows per subplot, sun-dried, and converted to kg/ha.

Grain Yield (kg/ha): Grains were weighed after threshing, cleaning, and sun drying.

Harvest Index (HI): Calculated as (Economic Yield / Biological Yield) × 100.

Correlation and Regression: Conducted analyses to assess relationships between yield and its components in mung bean varieties.

partial budget analysis

An economic analysis was conducted using partial budget analysis following the methods described by CIMMYT 1988 [18]. All variable costs were recorded and used for the analysis.

Data analysis

Before conducting the data analyses, we evaluated the assumptions related to data distribution and performed tests for multivariate normality, homogeneity of covariance matrices, and linearity. The Multivariate Analysis of Variance (MANOVA) analysis was carried out using SAS version 9.34 [18], following the procedures described by Gomez and Gomez (K.A. Gomez and A.A. Gomez, 1984). Interpretations were then derived from the results. To compare the means of significant treatments, we applied a 5% probability level and used the Tukey's HSD).

RESULTS

Soil analysis results

Table 2, presents soil physical and chemical properties of an experimental site before and after the harvest of a mung bean crop in two consecutive years, 2022 and 2023.

Table 2: Soil physical and chemical properties of the experimental site before and after crop harvest

**Cropping year**	**Soil test**	Physical properties (%)			PH(1:2.5 H2O)	OC (%)	Total N (%)	P(ppm)	S (ppm)	B(ppm)	CEC [cmol(+)kg-1]
		**Sand**	**Silt**	**Clay**							
**2022**	**Before planting**	50	18	32	5.63	2.8	0.06	27.25	18.25	0.57	22.22
	**After harvesting**	49.62	18.1	32.29	5.8	3.5	0.14	29.92	20.49	0.66	22.78
**2023**	**Before planting**	49.62	18.1	32.29	5.8	3.5	0.14	29.92	20.49	0.66	22.88
	**After harvesting**	50	20.3	29.7	6	3.64	0.17	30.7	20.6	0.8	29

Key Agronomic and Physiological components

Table 3: Main and interaction effects on the following variables: nodule number per plant, days to flowering, plant height, number of branches, fresh weight, dry matter, leaf area index, days to 90% maturity, hundred seed weight, biological yield, grain yield, and harvest index

Treatment	Nodule number per plant	Days to flowering	Plant height (cm)	Number of branches	Fresh weight (gm) per plant	Dry matter (gm) per plant	Leaf area index	Days to 90% maturity	Hundred seed weight(g)	Biological yield (tone /ha)	Grain yield (tone /ha)	Harvest index
**Cropping year(CR)**												
2022	8.8	59.5^{b}	52.23^{b}	5.43^{b}	27.2	2.3b	0.27^{b}	78.5^{b}	50.63^{b}	4.23	1.58^{b}	0.443^{b}
2023	8.4	63.33^{a}	58.2^{a}	5.6^{a}	28.6	2.6a	0.32^{a}	79.6^{a}	55.2^{a}	4.25	1.7^{a}	0.521^{a}
**LSD (5%)**	**1.8**	**3.91**	**6.3**	**0.9**	**ns**	**1.1**	**0.26**	**3.6**	**3.6**	**ns**	**0.45**	**0.2**
**CV(%)**	**8.5**	**4.09**	**6.5**	**9.4**	**4.9**	**1.3**	**0.16**	**8.6**	**7.8**	**2.1**	**0.8**	**0.35**
**Varieties (V)**	**ns**											
NVL-1	8.38^{b}	45.67^{b}	52.2^{bc}	5.4^{c}	29.6^{b}	4.6^{b}	0.33^{b}	78.4^{b}	55.93^{b}	4.27^{b}	1.76^{b}	0.521^{B}
N-26	8.45^{b}	44.5^{b}	53.27^{b}	5.6^{b}	32.6^{a}	52^{a}	0.34^{b}	79.76^{a}	57.0^{a}	4.42^{a}	1.95^{a}	0.536^{a}
Arkebe	9.6^{a}	50.6^{a}	58.53^{a}	5.97^{a}	28.6^{b}	4.8^{b}	0.38^{a}	78.9^{a}	55.2^{b}	4.099^{c}	1.74^{b}	0.526^{a}
**LSD (5%)**	**1.94**	**4.7**	**6.8**	**1.5**	**3.5**	**1.5**	**0.14**	**6.2**	**3.8**	**2.2**	**0.48**	**0.12**
**NPSB (kg/ha)**	**9.6**	**6.4**	**9.3**	**12.5**	**9.8**	**2.6**	**0.23**	**11**	**8.4**	**1.3**	**0.83**	**0.4**
0	8.38^{d}	45.67^{c}	52.2^{c}	5.1^{c}	13.3^{e}	2.5cd	0.29^{bc}	78.4^{d}	47.78^{d}	3.37^{cd}	1.20^{d}	0.231^{d}
25	9.6^{c}	60.6^{b}	58.53^{b}	5. 27^{c}	18.3^{b}	2.7c	0.29^{bc}	78.9^{c}	52.4^{c}	3.79^{c}	1.28^{d}	0.301^{C}
50	14.7^{b}	61.5^{a}	62.5^{a}	5.6^{b}	19.6^{b}	2.8c	0.32^{b}	80.76^{b}	55.4^{bc}	4.05^{b}	1.48^{c}	0.341^{C}
75	21.4^{a}	60^{b}	60.4^{a}	6.77^{a}	26^{a}	4.8^{ab}	0.35^{b}	80.3^{b}	54.73^{ba}	4.3^{a}	1.62^{b}	0.47^{b}
100	20.77^{a}	63^{a}	62.5^{a}	6.0^{a}	32.6^{a}	5.4^{a}	0.48a	84.4^{a}	57.6^{a}	4.4^{a}	1.94^{a}	0.54^{a}
**LSD (5%)**	**2.5**	**4.9**	**7.2**	**1.7**	**7.6**	**1.9**	**0.18**	**6.9**	**4.2**	**1.4**	**0.51**	**0.23**
**CV(%)**	**9.4**	**6.8**	**9.9**	**15.5**	**13.7**	**3.1**	**0.36**	**12.6**	**8.9**	**1.9**	**0.78**	**0.57**
	**P statistics**											
**CR**	ns	0.0033	0.022	<.0001	ns	0.023	0.04	0.0601	0.024	ns	0.002	0.034
**Varieties**	0.004	<.0001	0.1456	<.0003	0.008	0.015	0.026	<.0001	0.013	0.04	0.024	0.001
**NPSB (kg/ha)**	0.023	<.003	0.0431	0.0134	0.028	0.006	0.011	0.026	0.006	0.011	0.025	<.0001
**CR* V**	ns	ns	ns	ns	ns	ns	ns	ns	ns	ns	ns	ns
**CR* Fertilizer**	ns	ns	ns	ns	ns	ns	ns	ns	ns	ns	ns	ns
**V* Fertilizer**	ns	ns	ns	ns	ns	ns	0.004	ns	0.021	0.005	0.032	ns
**CR* V* NPSB**	ns	ns	ns	ns	0.001	0.021	0.0023	ns	ns	ns	ns	0.001

Letters in the same vertical column indicate no significant differences ($p \leq 0.05$, 0.01, or 0.001). CV is the coefficient of variation, LSD denotes significant differences, and ns means non-significant.

Effects on yield and yield traits of mung bean

Table 4 highlights the interaction effects of cropping year, varieties, and NPSB fertilizer rates on mung bean yield and components. Significant variations reveal that the combination of these factors influences performance, emphasizing the complexity of mung bean production.

Table 4. Interaction effects of varieties and NPSB fertilizer rates on the yield and yield components of mung bean varieties, averaged over two years (2022 and 2023).

Variety Type	Fertilizer Level (kg/ha)	Number of pods per plant	Number of seed per pod	Number of seed per plant	Hundred seed weight(g)	Biological yield(tone /ha)	Grain yield(tone /ha)
NVL-1	0	26.5^{hf}	10.5 ^{ef}	278.56^{gh}	49.9^{igh}	3.5742^{efg}	1.08e^{h}
NVL-1	25	28.4^{ef}	11.3^{fh}	299.5^{ih}	51.2^{ih}	3.397.1^{fg}	1.84^{ih}
NVL-1	50	33.0^{ef}	10.53^{fe}	338.66^{egh}	52.59^{fgh}	3.860^{efg}	1.28^{feh}
NVL-1	75	39.77^{bd}	12.33^{ed}	464.5^{fg}	50.67^{edcf}	4.1691^{abc}	1.52^{bc}
NVL-1	100	48.54^{ba}	12.5^{cb}	660.6^{ab}	55.93^{bac}	4.2603^{ab}	1.76^{b}
N-26	0	29.34^{h}	11.5^{fg}	288.65^{ifg}	47.78^{fh}	3.3685^{hgi}	1.20^{h}
N-26	25	33.45^{e}	12.987^{cd}	339.6^{gh}	52.4^{ecd}	3.7824^{efg}	1.28^{fgh}
N-26	50	38.56^{ce}	12.77 ^{ac}	472.4^{dfeh}	55.4^{ba}	4.0432^{bc}	1.48^{cd}
N-26	75	45.17^{ba}	14^{b}	560.2^{c}	54.73^{bc}	4.2597^{ba}	1.6^{b}
N-26	100	51.8^{a}	15.8^{a}	769.7a	57.0^{a}	4.4114^{a}	1.94^{a}
Arkebe	0	26.77^{fh}	8.9^{h}	267.1^{ij}	46.3^{ij}	3144.3^{ik}	1.27^{gh}
Arkebe	25	32.64^{ef}	11.12^{fe}	322.45^{ihj}	47.77^{fgh}	3.5588^{gh}	1.285 ^{fdh}
Arkebe	50	34.65^{de}	12.6^{cd}	414.0^{gfh}	51.6^{dfe}	3.7887^{fgh}	1.38^{de}
Arkebe	75	42.34^{bd}	12.48^{cb}	536.67^{bdc}	50.63^{bed}	4.1248^{cde}	1.58^{cd}
Arkebe	100	46.47^{ba}	14.5a^{cb}	492.44^{dce}	55.2^{bac}	4.0985^{bde}	1.7^{acb}
**LSD (5%)**		**6.2**	**2.3**	**65**	**3.6**	**2.085**	**0.87**
**CV(%)**		**10.7**	**11.5**	**15.5**	**8.5**	**12.2**	**3.8**

Letters in the same vertical column indicate no significant differences ($p \leq 0.05$, 0.01, or 0.001). CV is the coefficient of variation, LSD denotes significant differences, and ns means non-significant.

Table 5: Interaction effects of two years, varieties, and NPSB fertilizer rates on mung bean varieties concerning fresh weight (g per plant), dry matter (g per plant), leaf area index (LAI), and harvest index.

**Treatment combinations**		**Fresh weight (gm) per plant**		**Dry matter (gm) per plant**		**LAI**		**Harvest index**	
**Varieties**	**NPSB rates (kg/ha)**	**2022**	**2023**	**2022**	**2023**	**2022**	**2023**	**2022**	**2023**
NVL-1	0	11.3^{e}	15.4^{d}	1.9^{d}	2.1^{d}	0.22^{d}	0.25^{c}	0.211^{d}	0.232^{d}
	25	15.6^{d}	21.5^{ab}	2.3^{c}	2.4^{c}	0.23^{d}	0.25^{c}	0.311^{bc}	0.355^{c}
	50	18.6^{c}	19.7^{ab}	2.6^{b}	2.4^{c}	0.28$_{b}$	0.283^{bc}	0.388^{ab}	0.398^{ab}
	75	23.6^{ab}	28.5^{a}	3.2^{b}	4.3^{b}	0.27^{b}	0.35^{b}	0.343^{ba}	0.384^{ab}
	100	29.6^{a}	30.3^{a}	4.6^{a}	4.8^{ab}	0.32^{a}	0.45^{a}	0.521^{a}	0.571^{a}
N-26	0	13.3^{e}	16.8^{d}	2.2^{cd}	2.5^{c}	0.24^{c}	0.29^{bc}	0.211^{d}	0.256^{c}
	25	18.3^{b}	23.8^{ab}	2.4^{c}	2.7^{cb}	0.25^{c}	0.29^{bc}	0.301^{b}	0.365^{bc}
	50	19.6^{b}	20.7^{ab}	2.7^{b}	2.8^{cb}	0.29^{b}	0.32^{b}	0.341^{b}	0.386^{ba}
	75	26^{a}	27.5^{a}	3.7^{ab}	4.8^{ab}	0.27^{b}	0.35^{b}	0.465^{ab}	0.495^{ab}
	100	32.6^{a}	34.6^{a}	4.5^{a}	5.5^{a}	0.35^{a}	0.48a	0.54^{a}	0.589^{a}
Arkebe	0	11.0^{e}	15.3^{d}	1.7^{d}	1.9^{d}	0.22^{d}	0.24^{d}	0.248^{d}	0.248^{c}
	25	16.6^{d}	19.5^{ab}	2.4c	2.5^{c}	0.21^{d}	0.23^{d}	0.321^{b}	0.353^{c}
	50	18.6^{b}	19.6^{ab}	2.6^{b}	2.6^{c}	0.27^{b}	0.30$_{b}$	0.341^{b}	0.364^{b}
	75	27.2^{a}	28.5^{a}	3.2^{b}	4.2^{b}	0.29^{b}	0.37^{b}	0.465^{ab}	0.483^{ab}
	100	28.6^{a}	30.6^{a}	4.8^{a}	5.2^{a}	0.38^{a}	0.46^{d}	0.526^{a}	0.554^{a}

Letters in the same vertical column indicate no significant differences ($p \leq 0.05$, 0.01, or 0.001). CV is the coefficient of variation, LSD denotes significant differences, and ns means non-significant.

DISCUSSIONS

Soil Physico-chemical Properties

The study revealed significant changes in soil properties post-harvest (Table 2). Sand content decreased slightly, while silt content increased in both years, suggesting reduced soil coarseness due to root activity and organic matter decomposition [20]. Clay content remained stable, preserving soil structure.

Soil pH increased after NPSB fertilizer application, rising from 5.63 to 5.8 in 2022 and to 6.0 in 2023, indicating its role in neutralizing soil acidity [20]. Organic carbon (OC) content increased from 2.8% to 3.5% in 2022 and further to 3.64% in 2023, reflecting mung bean contributions to organic matter [21]. Total nitrogen rose from 0.06 ppm to 0.14 ppm in 2022 and to 0.17 ppm in 2023, attributed to atmospheric nitrogen fixation by mung beans [22;23].

Available phosphorus (Avail P) increased from 27.25 ppm to 29.92 ppm in 2022 and to 30.7 ppm in 2023, linked to enhanced phosphorus availability from fertilizer (Ro et al., 2022). Sulfur (S) content rose from 18.25 ppm to 20.49 ppm in 2022 and to 20.6 ppm in 2023, consistent with NPSB fertilizer use [24]. Boron (B) content increased from 0.57 ppm to 0.66 ppm in 2022 and to 0.8 ppm in 2023 due to fertilizer application [25]. Cation exchange capacity (CEC) improved significantly from 22.22 to 22.78 cmol(+)/kg in 2022 and to 29 cmol(+)/kg in 2023, enhancing soil nutrient retention.

Nodule Number per Plant

No significant differences in nodule numbers were observed between 2022 and 2023 (Table 3). However, significant varietal differences were noted, with Arkebe exhibiting the highest nodule count (9.6) compared to NVL-1 (8.38) and N-26 (8.45), highlighting genetic variability in nodulation capacity [26]. Nodule numbers increased significantly with higher NPSB fertilizer rates, peaking at 75 kg/ha (21.4) and 100 kg/ha (20.77), suggesting improved phosphorus availability enhances nodulation [26;27].

Days to Flowering

Significant variations in flowering duration were observed between the two years, with delayed flowering in 2023 (63.33 days) compared to 2022 (59.5 days), likely due to environmental factors such as temperature and rainfall [28]. Among genotypes, Arkebe exhibited the longest flowering duration (50.6 days), followed by NVL-1 (45.67 days) and N-26 (44.5 days), reflecting genetic differences in phenological responses. Higher NPSB application rates delayed flowering, with 50 kg/ha (61.5 days) and 100 kg/ha (63 days) showing the longest durations, consistent with findings by [29].

Plant Height

Plant height was significantly greater in 2023 (58.2 cm) than in 2022 (52.23 cm), likely due to improved growing conditions or enhanced soil fertility (Table 3). Among varieties, Arkebe was the tallest (58.53 cm), followed by N-26 (53.27 cm) and NVL-1 (52.2 cm), demonstrating genetic variability in plant architecture [5;30]. Phosphorus in NPSB fertilizer improved root growth, nutrient uptake, and drought tolerance, contributing to increased plant height [31; 32].

Number of Branches per Plant

Branching increased significantly in 2023 (5.6) compared to 2022 (5.43), with Arkebe exhibiting the highest branching capacity (5.97). Branching peaked at 6.77 with 75 kg/ha NPSB application, indicating balanced nutrient application enhances branching. This aligns with [33], who found increased nutrient availability promotes branch formation and vigorous growth.

Days to 90% Maturity

The 2023 season required slightly more time to reach maturity compared to 2022. Varietal differences were significant, with N-26 and Arkebe taking longer than NVL-1. Higher NPSB application rates delayed maturity, with 100 kg/ha resulting in the longest duration (84.4 days) (Table 3). This delay is likely due to nitrogen promoting vegetative growth, postponing reproductive development, consistent with findings by [34].

Interaction effects of varieties, and NPSB fertilizer rates on mung bean

Effects on the Number of Pods per Plant

Combined data from 2022 and 2023 revealed a significant interaction between variety and fertilizer level ($p \leq 0.05$), as shown in Tables 3 and 4. The N-26 variety outperformed NVL-1 and Arkebe at 100 kg/ha, though differences were minimal at lower fertilizer rates. This superior performance is likely due to better nutrient uptake, consistent with [35] findings. The study aligns with [36] emphasis on trait selection in breeding and [37] focus on matching varieties with appropriate fertilizer management. N-26 produced the highest number of pods (51.8) at 100 kg/ha, supporting [38] research on nitrogen's role in legume pod development.

Number of Seeds per Pod

The number of seeds per pod increased with fertilizer application, but the extent varied by variety. N-26 recorded the highest number (15.8 at 100 kg/ha; Table 4), indicating superior reproductive efficiency under high nitrogen conditions. These results highlight the importance of variety-fertilizer combinations for yield optimization, consistent with crop science principles [39]. The findings support [40] and [41], in emphasizing multifactorial management for sustainability.

Seeds per Plant

This metric combines pods per plant and seeds per pod, providing a holistic view of yield. N-26 consistently outperformed other varieties, especially at higher fertilizer rates (Table 4), demonstrating its potential for high-yield systems. The strong response of N-26 to fertilizer aligns with [42] and [40] While Arkebe showed stable performance across fertilizer rates, its yield potential remained lower than N-26 and NVL-1.

Hundred Seed Weight (g)

N-26 exhibited higher seed weights across all fertilizer rates, particularly at 100 kg/ha (57.0 g; Table 4). Arkebe recorded the lowest weight at 0 kg/ha (46.3 g) but improved significantly at 100 kg/ha (55.2 g), indicating greater responsiveness to fertilizer than NVL-1, consistent with [43]. Increasing fertilizer rates generally enhanced seed weight across all varieties, aligning with [44], who reported that NPSB fertilization boosts seed weight.

Biological Yield (t/ha)

N-26 attained the highest biological yield at 100 kg/ha (4.4114 t/ha). NVL-1 showed a moderate increase (3.5742 t/ha to 4.2603 t/ha), while Arkebe exhibited a more significant response (3.1443 t/ha to 4.0985 t/ha). These results emphasize the importance of optimal nutrient management, as 100 kg/ha was highly effective for N-26 [45;46]. N-26 demonstrates strong potential for high productivity, while Arkebe is less resilient under low-input conditions, likely due to environmental factors [23; 47].

Grain Yield (t/ha)

N-26 achieved the highest grain yield at 100 kg/ha (1.94 t/ha), followed by Arkebe (1.7 t/ha) and NVL-1 (1.76 t/ha; Table 4). These results align with [48], indicating treatment effects rather than random variation. Grain yield increased with fertilizer application, but the response was nonlinear. For instance, NVL-1 showed a yield decline at 50 kg/ha (1.28 t/ha) compared to 25 kg/ha (1.84 t/ha), suggesting a potential threshold effect or nutrient imbalance at intermediate rates. Optimal fertilizer application likely provided the best nutrient balance for growth, maximizing yield [49]. These findings underscore the importance of proper fertilizer use for enhancing crop yield [50]. Varieties responded differently to fertilizer rates, with N-26 demonstrating greater efficiency in converting nutrients into grain yield.

Interaction of years, varieties, NPSB rates on mung bean metrics

Effects on fresh weight

The three ways-interaction effects of years, varieties, and NPSB rates are highly significant ($p \leq 0.001$), as shown in Tables 3 and 5. Variety N-26 consistently yields higher fresh weights than NVL-1 and Arkebe across most NPSB rates. For example, in 2023, N-26 at 100 kg/ha recorded a fresh weight of 34.6 gm, compared to 30.3 gm for NVL-1 and 30.6 gm for Arkebe, aligning with findings by [51]. Arkebe, though showing lower fresh weights at lower NPSB rates, performs better at higher rates (75 and 100 kg/ha), suggesting greater responsiveness to increased fertilization. Across all varieties, higher NPSB rates generally lead to increased fresh weight. For instance, NVL-1's fresh weight rose from 11.3 gm at 0 kg/ha to 29.6 gm at 100 kg/ha in 2022, and

from 15.4 gm to 30.3 gm in 2023. The highest fresh weights were consistently observed at 100 kg/ha for all varieties in both years, indicating this level as optimal for maximizing fresh weight [52].

Effects on dry weight

The interaction effects of years, varieties, and NPSB rates were highly significant ($p \leq 0.001$), as shown in Tables 3 and 5. N-26 consistently outperformed NVL-1 and Arkebe at higher NPSB rates (e.g., 5.5 gm/plant at 100 kg/ha in 2023). Arkebe, with the lowest yields at 0 kg/ha, responded well to higher NPSB rates, nearly matching N-26 at 100 kg/ha, indicating genetic differences in nutrient use efficiency [53]. Increasing NPSB rates generally boosted dry matter yields across varieties. For instance, NVL-1 increased from 1.9 gm/plant (0 kg/ha) to 4.6 gm/plant (100 kg/ha) in 2022, with similar trends for N-26 and Arkebe. Balanced fertilization (NPSB: nitrogen, phosphorus, sulfur, boron) significantly enhances growth and biomass [54]. Yields were higher in 2023 than 2022, likely due to environmental factors like rainfall and temperature, which influence nutrient availability [55].

Effects on leaf area index

The LAI values vary significantly across varieties, NPSB levels, and years, indicating a complex interaction. For instance, NVL-1's LAI at 100 kg/ha NPSB increased from 0.32 in 2022 to 0.45 in 2023, suggesting year-specific environmental or management influences. Generally, LAI increases with higher NPSB levels, peaking at 100 kg/ha, reflecting improved nutrient availability for photosynthesis and growth. However, the response magnitude varies by variety and year. N-26 showed a smaller LAI increase (0.27 to 0.35) from 75 kg/ha to 100 kg/ha in 2022 compared to Arkebe (0.29 to 0.38). Arkebe consistently achieved the highest LAI at 100 kg/ha NPSB, indicating superior responsiveness, while NVL-1 and N-26 exhibited lower values, likely due to genetic differences in nutrient utilization. Increased LAI is linked to enhanced nutrient availability, supporting physiological processes [56], consistent with studies emphasizing nutrient importance in plant development [4; 57].

Effects on Harvest index

NVL-1 and N-26 demonstrate a significant increase in Harvest Index (HI) with rising NPSB rates, peaking at 100 kg/ha in both years (NVL-1: 0.521 in 2022, 0.571 in 2023; N-26: 0.54 in 2022, 0.589 in 2023). Arkebe also responds positively but shows a less pronounced HI increase, especially at lower NPSB rates (25 and 50 kg/ha). Across all varieties and years, HI consistently rises with higher NPSB rates, reaching its maximum at 100 kg/ha, indicating improved biomass partitioning into economic yield, aligning with [24;29]. The lowest HI values occur at 0 kg/ha NPSB, highlighting the critical role of fertilization in resource allocation for grain production. Higher HI values in 2023 compared to 2022 suggest more favorable environmental conditions, potentially due to variations in rainfall, temperature, or other agronomic factors [58].

Correlations and regressions

This study found a strong positive correlation between fertilizer application and nodule number per plant ($R^2 = 0.73$, $p < 0.01$), highlighting that increased fertilizer enhances nodule formation, essential for nitrogen fixation, consistent with [59]. Similarly, fertilizer application strongly correlated with grain yield ($R^2 = 0.95$, $p < 0.001$), indicating its role in boosting yield and economic returns, aligning with [60]. A moderate positive correlation was observed between seeds per pod and hundred seed weight ($R^2 = 0.85$, $p < 0.01$), suggesting that more nodules improve seed weight, a critical yield factor [59]. Additionally, plant height and branch number showed a high positive correlation ($R^2 = 0.84$, $p < 0.05$), implying taller plants with more branches may enhance yield and reduce weather-related risks.

Partial budget analysis

This study assessed the efficiency and economic viability of mung bean production over two consecutive cropping years, comparing yields between experimental and farmers' fields. It investigated the impact of different fertilizers, crop varieties, and application methods on costs and benefits. Utilizing a multi-criteria decision-making approach, the study identified the most cost-effective treatments based on economic performance, distinguishing between dominating and non-dominated options.

The results revealed that the N-26 variety, fertilized with NPSB at 100 kg/ha, generated the highest net benefit, yielding 47,704.17 ETB/ha in 2022 and 49,856.85 ETB/ha in 2023. These findings are

consistent with those of [61], who also demonstrated that optimal NPSB fertilizer application enhances the economic performance of mung bean production.

The study provides valuable insights for policymakers, farmers, and stakeholders, emphasizing best practices for cost-effective mung bean production, such as optimal fertilizer application and variety selection. The recommendations, which focus on minimum return rates, can guide decision-making to maximize investment returns. Furthermore, the methodology and findings of this study can be adapted to other regions, offering a framework for shaping sustainable agricultural policies and practices.

CONCLUSIONS

The results indicated that both the fertilizer and variety significantly influenced soil pH, available boron, and organic carbon content. The optimal fertilizer rate was found to be 100 kg/ha, which resulted in the highest growth and yield parameters. Among the varieties, N-26 excelled in branches, height, seed yield, and harvest index over two years. In 2023, N-26 with 100 kg/ha produced the highest grain yield of 1.94 t/ha, while Arkebe with no fertilizer yielded the lowest at 1.27 t/ha. The N-26 variety also achieved the greatest net benefit, yielding 47,704.17 ETB/ha in 2022 and 49,856.85 ETB/ha in 2023. It is advisable to use 100 kg/ha of NPSB fertilizer for the N-26 variety. Further research is needed to confirm these results over multiple years and with additional varieties and fertilizer rates.

Authorship contribution statement

In this project, Daniel led the writing and editing process while contributing to the conceptual framework. Oumer gathered data and managed fieldwork, ensuring solid empirical support. Hiwot analyzed the data, drafted the original manuscript, refined the methodology, and participated in the review to improve clarity. Francis provided editing and co-supervision, offering valuable insights. Together, they formed a cohesive team, each contributing unique strengths.

Declaration of generative AI and AI-assisted technologies

During the preparation of this work the authors used QuillBot in order to improve language and readability. After using this QuillBot, the authors reviewed and edited the content as needed and take full responsibility for the content of the publication.

Declaration of Competing Interest

The authors report no declarations of interest.

Acknowledgments

We express our gratitude to Wachemo University for providing us with the necessary field and laboratory facilities. Additionally, we acknowledge the financial support received from the Dalocha Woreda Silete zone, which enabled us to carry out this work.

REFERENCES

1. Kedir, U. (2023). Advances in Crop Science and Technology Effect of NPS Fertilizer Rates on Growth, Yield and Yield components of Mungbean (Vigna radiata L .) variety at low land of East Shawa , Ethiopia. 11(2). https://doi.org/10.4172/2329-8863.1000558
2. Favero, V. O., Carvalho, R. H., Motta, V. M., Leite, A. B. C., Coelho, M. R. R., Xavier, G. R., Rumjanek, N. G., & Urquiaga, S. (2021). Bradyrhizobium as the Only Rhizobial Inhabitant of Mung Bean (Vigna radiata) Nodules in Tropical Soils: A Strategy Based on Microbiome for Improving Biological Nitrogen Fixation Using Bio-Products. Frontiers in Plant Science, 11(January), 1–16. https://doi.org/10.3389/fpls.2020.602645
3. Wilbur, J. (2022). , 2022 Blacksburg, Virginia Keywords: mungbean, Senegal, food security, plant protein, meat alternatives, sustainable agriculture.
4. Bean, M., Polyphenols, L. B., & Benefits, H. (2019). Mung Bean (Vigna radiata L.): Bioactive Polyphenols, Polysaccharides, Peptides, and Health Benefits. 1–28.
5. Dikir, W., & Garkebo, H. (2022). Agronomic and other Traits of Mung Bean (Vigna Radiata L .) Response to Different Rates of Phosphorus Fertilizer and Row Spacing for Better Grain Yield Production in Silte Southern Ethiopia. July. https://doi.org/10.23880/oajar-16000287
6. Assefa, Z. B., Dinku, A. M., & Jemal, A. M. (2022b). Value chain analysis of Mung Bean (Vigna radiata L. Wilczek thrive) in Kalu Woreda, Ethiopia. Agriculture and Food Security, 11(1), 1–14. https://doi.org/10.1186/s40066-022-00393-2
7. Baza, M., Shanka, D., & Bibiso, M. (2022). Agronomic and economic performance of mung bean (Vigna radiata L.) varieties in response to rates of blended NPS fertilizer in Kindo Koysha district, Southern Ethiopia. Open Life Sciences, 17(1), 1053–1063. https://doi.org/10.1515/biol-2022-0461
8. Yimer, Z. (2022). Case study report on improved mung bean technology demonstration under. April. https://doi.org/10.33545/2664844X.2022.v4.i1a.63
9. Karavidas, I., Ntatsi, G., Vougeleka, V., Karkanis, A., & Ntanasi, T. (2022). Agronomic Practices to Increase the Yield and Quality of Common Bean Agronomic Practices to Increase the Yield and Quality of Common Bean (Phaseolus vulgaris L .): A Systematic Review. February. https://doi.org/10.3390/agronomy12020271
10. Elias, E., Okoth, P. F., Stoorvogel, J. J., Berecha, G., Mellisse, B. T., Mekuriaw, A., Gebresamuel, G., Selassie, Y. G., Biratu, G. K., & Smaling, E. M. A. (2023). Cereal

yields in Ethiopia relate to soil properties and N and P fertilizers. Nutrient Cycling in Agroecosystems, 126(2–3), 279–292. https://doi.org/10.1007/s10705-023-10291-z

11. Ryti, R. (1965). On the determination of soil pH. *Agricultural and Food Science*, *37*(1), 51-60.
12. Mylavarapu, R., Sikora, F. J., & Moore, K. P. (2014). Walkley-Black Method. *Soil test methods from the Southeastern United States*, *158*.
13. Hicks, T. D., Kuns, C. M., Raman, C., Bates, Z. T., & Nagarajan, S. (2022). Simplified method for the determination of total kjeldahl nitrogen in wastewater. *Environments*, *9*(5), 55.
14. Wall, L. L., Gehrke, C. W., & Suzuki, J. (1980). An automated turbidimetric method for total sulfur in plant tissue and sulfate sulfur in soils. *Communications in Soil Science and Plant Analysis*, *11*(11), 1087-1103.
15. Huluka, G., & Miller, R. (2014). Particle size determination by hydrometer method. *Southern Cooperative Series Bulletin*, *419*, 180-184.
16. Kitsopoulos, K. P. (1999). Cation-exchange capacity (CEC) of zeolitic volcaniclastic materials: applicability of the ammonium acetate saturation (AMAS) method. *Clays and clay minerals*, *47*(6), 688-696.
17. Miller, J. J., & Curtin, D. (2006). Electrical conductivity and soluble ions. *Soil sampling and methods of analysis*, *2*.
18. Roth, S., & Hyde, J. (2002). Partial bdgeting for agricultural businesses. College of Agricultural Sciences, Agricultural Research and Cooperative Extension, The Pennsylvania State University. CAT UA366 5M2/02ps4486. 1–8.
19. Wang, C. (1992). *Nonlinear multivariate analysis of variance with application to event-related potential studies*. University of California, Los Angeles
20. Abdulkadir, M. O., & Vision, W. (2017). Chapter two: Soil physical properties Mohamed Osman Abdulkadir--B. Sc ., in Agriculture. October 2016.
21. Diatta, A. A., Thomason, W., Seleiman, M., Bassène, C., Battaglia, M., Manga, A. G. B., Abaye, O., Babur, E., Uslu, Ö., Min, D., Mbow, C., & Filho, J. F. D. C. L. (2023). Integrated use of organic amendments increased mungbean (Vigna radiata (L .) Wilczek) yield and its components compared to inorganic fertilizers. September, 1–15. https://doi.org/10.1002/uar2.20048
22. Khan, H., Ali, W., Shah, Z., Ur, H., Taj, A., & Alatalo, J. M. (2022). Heliyon Coupling phosphate-solubilizing bacteria (PSB) with inorganic phosphorus fertilizer improves mungbean (Vigna radiata) phosphorus acquisition , nitrogen fi xation , and yield in alkaline-calcareous soil. Heliyon, 8(March), e09081. https://doi.org/10.1016/j.heliyon.2022.e09081
23. Ro, S., Roeurn, S., Sroy, C., & Prasad, P. V. V. (2023). Agronomic and Yield Performance of Maize-Mungbean Intercropping with Different Mungbean Seed Rates under Loamy Sand Soils of Cambodia.

24. Mota, F. M., Balla, D. S., & Doda, M. B. (2021a). Response of Mung Bean Varieties (Vigna radiata L .) to Application Rates and Methods of Blended NPS Fertilizer at Humbo. 2021.
25. Patgiri, P., Swami, S., Singh, T. D., Sri, M., & Charan, S. (2023). Boron Availability as Influenced by Soil Physico-chemical Properties (Issue August).
26. Sulieman, S., & Tran, L. S. P. (2015). Phosphorus homeostasis in legume nodules as an adaptive strategy to phosphorus deficiency. *Plant Science*, 239, 36–43.
27. Tekulu, K., Taye, G., & Assefa, D. (2020). Heliyon Effect of starter nitrogen and phosphorus fertilizer rates on yield and yield components, grain protein content of groundnut (Arachis Hypogaea L .) and residual soil nitrogen content in a semiarid north Ethiopia. Heliyon, 6(August 2019), e05101. https://doi.org/10.1016/j.heliyon.2020.e05101
28. Lobell, D. B., Hammer, G. L., McLean, G., Messina, C., Roberts, M. J., & Schlenker, W. (2015). The critical role of extreme heat for maize production in the United States. *Nature Climate Change*, 5(5), 497–501.
29. Mota, F. M., Balla, D. S., & Doda, M. B. (2021b). Response of mung bean varieties (Vigna radiata L.) to application rates and methods of blended NPs fertilizer at humbo. *International Journal of Agronomy*, *2021*(1), 3786720.
30. Kaysha, K., Shanka, D., Bibiso, M., & Moral, M. T. (2020). Performance of mung bean (Vigna radiata L .) varieties at different NPS rates and row spacing at Kindo Koysha district , Southern Ethiopia. Cogent Food & Agriculture, 6(01), 1–12. https://doi.org/10.1080/23311932.2020.1771112
31. Id, Z. Y., Guo, W., Xiao, H., Liang, J., Hao, X., Dong, N., Leng, T., Wang, Y., Wang, Q., & Yin, F. (2018). Nitrogen , phosphorus , and potassium fertilization to achieve expected yield and improve yield components of mung bean. 1–17. https://doi.org/10.1371/journal.pone.0206285
32. Oromia, S., Olika, G. I., Ayana, D. T., & Daba, N. A. (2024). Yield Components and Yield of Common Bean (Phaseolus vulgaris L .) Varieties as Influenced by Rates of Phosphorus. 13(6), 249–259
33. Tirfessa, T., Jalata, Z., & Lamessa, K. (2022). Influence of blended NPSB fertilizer on nodulation and yield performance of Rhizobium inoculated common bean varieties in acidic soil western. All Life, 15(1), 1290–1303. https://doi.org/10.1080/26895293.2022.2156625
34. 34. Aslam, M., Khan, P., Imtiaz, M., & Depar, N. (2006). Effect of different methods and rates of phosphorus application in mungbean Effect of different methods and rates of phosphorus application. June 2014.
35. Walsh, K. B. (2024). Screening New Mungbean Varieties for Terminal Drought Tolerance.

36. Aklilu, M., & Abebe, T. (2021). Adaptation study of mung bean (Vigna radiata) varieties in tepi , south wester Adaptation study of mung bean (Vigna radiata) varieties in tepi , south wester Ethiopia
37. Li, X., Zhang, Y., & Wang, L. (2021). Optimizing nitrogen use efficiency in legume crops: Insights from genotype-by-environment interactions. *Agronomy Journal, 113*(4), 3456-3468.
38. Imran, Ì., Khan, A. A., Inam, I., & Ahmad, F. (2016). Yield and yield attributes of Mungbean (Vigna radiata L.) cultivars as affected by phosphorous levels under different tillage systems. *Cogent Food & Agriculture*, *2*(1), 1151982.
39. Penuelas, J., Coello, F., & Sardans, J. (2023). A better use of fertilizers is needed for global food security and environmental sustainability. Agriculture & Food Security, 1–9. https://doi.org/10.1186/s40066-023-00409-5
40. Abed, A., Al-shammary, G., Saleem, L., Al-shihmani, S., & Fern, J. (2024). Optimizing sustainable agriculture: A comprehensive review of agronomic practices and their impacts on soil attributes.
41. Rojas-ospina, A., & Zu, A. (2024). Journal of Open Innovation: Technology , Market , and Complexity Factors influencing environmental sustainability performance : A study applied to coffee crops in Colombia. 10(January).
42. Kassa, Y., Admasu, D., Tigabe, A., Abie, A., & Mamo, D. (2018). Participatory on farm evaluation of improved mungbean technologies in the low land areas of North Shewa Zone Amhara Region, Ethiopia. 10(August), 158–164. https://doi.org/10.5897/JAERD2018.0962
43. Kassa, Y., Abie, A., Mamo, D., & Ayele, T. (2023). Heliyon Exploring farmer perceptions and evaluating the performance of mung bean (Vigna radiata L) varieties in Amhara region , Ethiopia. Heliyon, 8(May 2022), e12525. https://doi.org/10.1016/j.heliyon.2022.e12525
44. Yin, Z., Guo, W., Xiao, H., Liang, J., Hao, X., Dong, N., ... & Yin, F. (2018). Nitrogen, phosphorus, and potassium fertilization to achieve expected yield and improve yield components of mung bean. *PloS one*, *13*(10), e0206285.
45. Baghlani, A. W., Alefzai, M., Hussainee, H., & Haidari, M. D. (2024). Influence of poultry manure level compared to nutrient- rich organic fertilizer on growth and yield of mung bean (Vigna radiate L .). 7(4), 348–358.
46. Tripathi, S., Kaur, A., Brar, A. S., Sekhon, K. S., Singh, S., & Malik, A. (2024). Optimizing yield and water productivity in summer mung bean (Vigna radiata L .) through crop residue management and irrigation strategies. 1–17.
47. Kassa, Y., Mamo, D., Abie, A., Tigabe, A., & Ayele, T. (2021). International Journal of Agricultural Extension scaling up of improved mung bean technology in the potential areas. 09(02), 135–141. https://doi.org/10.33687/ijae.009.02.3257
48. Kebede, Z. Y., & Kasim, B. (2024). Improved Mung Bean (Vigna radiate L .) Technology Demonstration and Promotion Under North-West Ethiopia. 8(3), 52–61.

49. 49. Noulas, C., Torabian, S., & Qin, R. (2023). Crop Nutrient Requirements and Advanced Fertilizer Management Strategies. 1–10.
50. Ghimirey, V., Chaurasia, J., Acharya, N., Marahatta, S., & Devkota, K. (2024). Journal of the Saudi Society of Agricultural Sciences Soil properties and yield of mung bean [vigna radiata (l .) wilczek] as influenced by tillage and phosphorous fertilizer management on sandy loam soil in Chitwan , Nepal. Journal of the Saudi Society of Agricultural Sciences, October. https://doi.org/10.1016/j.jssas.2024.10.001
51. Astutik, D., Yudhono, P., & Waluyo, S. (2017). The Growth of Two Cultivars Mung Bean under different Sweet Corn Shelter Density in Sandy Soil Coastal Area. *Ilmu Pertanian (Agricultural Science)*, *2*(3), 106-111.
52. Singh, D., Sharma, N. L., Singh, C. K., Yerramilli, V., Narayan, R., Sarkar, S. K., & Singh, I. (2021). Chromium (VI)-induced alterations in physio-chemical parameters, yield, and yield characteristics in two cultivars of mungbean (Vigna radiata L.). *Frontiers in plant science*, *12*, 735129.
53. Shiferaw, E. T., & Abewoy, D. (2023). Response of Mung Bean (Vignaradiata L.) Varieties to Phosphorus Fertilizer Rates Under Irrigation Condition. *Advances in Biochemistry*, *11*(4), 53-66.
54. Balaso, m. n. s. (2024). *evaluation of nutritional qualities and antinutritional contents in mungbean genotypes* (doctoral dissertation, mahatma phule krishi vidyapeeth).
55. Chauhan, Y. S., & Williams, R. (2018). Physiological and agronomic strategies to increase mungbean yield in climatically variable environments of Northern Australia. *Agronomy*, *8*(6), 83.
56. Alsamadany, H. (2022). Saudi Journal of Biological Sciences Physiological, biochemical and molecular evaluation of mungbean genotypes for agronomical yield under drought and salinity stresses in the presence of humic acid. Saudi Journal of Biological Sciences, 29(9), 103385. https://doi.org/10.1016/j.sjbs.2022.103385
57. Tehulie, N. S. (2020). Effects of NPS Fertilizer Rates on Growth , Yield and Yield Components of Mungbean [Vigna radiata (L .) Wilczek] Varieties under Irrigation at. 7(5), 217–233.
58. 58. Brezeanu, P. M., Brezeanu, C., Ambăruş, S., Străjeru, S., Robu, T., Cristea, T. O., & Călin, M. (2018, August). Grain legumes sustainable management and utilization for food, nutrition and environmental security. In *XXX International Horticultural Congress IHC2018: V International Symposium on Plant Genetic Resources and International 1297* (pp. 327-336).
59. Bam, R., Mishra, S. R., Khanal, S., Ghimire, P., & Bhattarai, S. (2022). Effect of biofertilizers and nutrient sources on the performance of mungbean at Rupandehi , Nepal. 10(February), 0–8.
60. Wabela, R., Abera, G., Lemma, B., & Gobena, A. (2024). Heliyon Effects of integrated fertilizer application on selected soil properties and yield attributes of common bean (Phaseolus vulgaris L .) on different soil types. 10(June).

61. Assefa, Z. B., Dinku, A. M., & Jemal, A. M. (2022a). Value chain analysis of Mung Bean (Vigna radiata L . Wilczek thrive) in Kalu Woreda , Ethiopia. Agriculture & Food Security, 1–14. https://doi.org/10.1186/s40066-022-00393-2

Figure Click here to access/download;Figure;Figures.docx

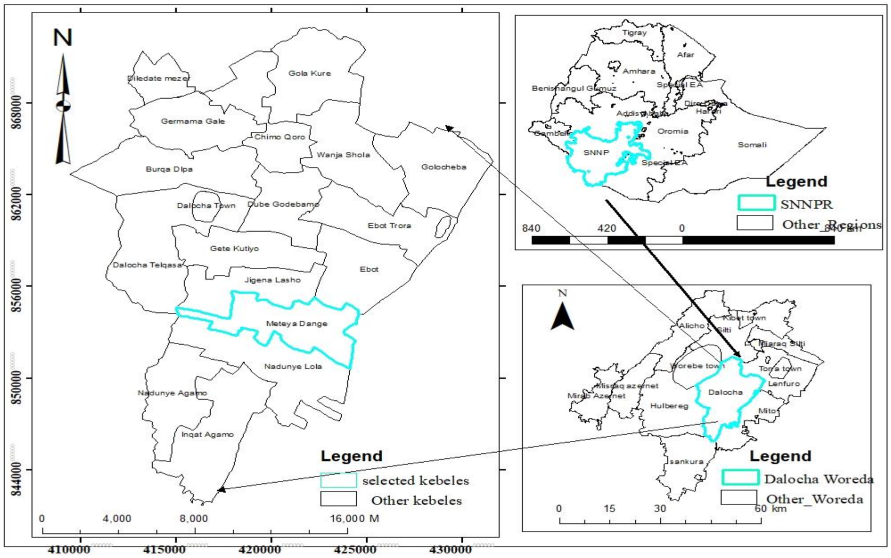

Figure 1:The geographical location of the experimental site.

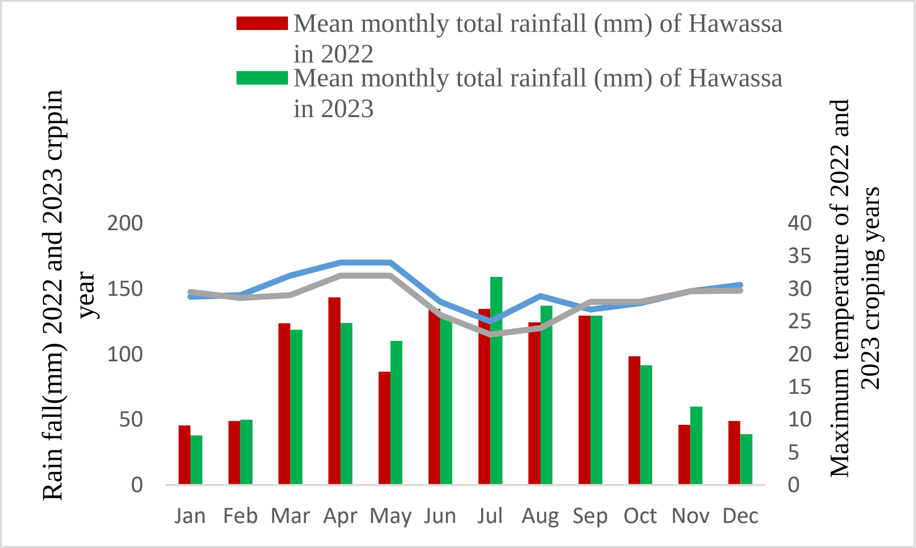

Figure 2:Weather data of cropping years’ s at Dalocha
