# Supplementary material for: Optimizing NPSB fertilizer rates for enhanced yield and yield components of mung bean (Vigna radiata L:) varieties
Source: PLoS One. 2025 Oct 24;20(10):e0321243. doi: 10.1371/journal.pone.0321243 (PMC12551827; doi:10.1371/journal.pone.0321243)
Supplement: Attachment — Submitted filename: General comments.docx [file pone.0321243.s002.docx]

**Comments to the Author/s**

Manuscript ID: PONE-D-25-10453

Manuscript title: **Optimizing NPSB Fertilizer Application Rates for Enhanced Yield and Yield Components of Mung Bean (Vigna Radiata L.) Varieties in the Dalocha District of Central Ethiopia**

**General comments**

The manuscript is relevant and well-written, with very few corrections required.

**Specific comments**

**Title**

- The title should be smart and specific, so I suggest changing it to “**Optimizing NPSB Fertilizer Rates for Enhanced Yield and Yield Components of Mung Bean (*Vigna radiata* L.) Varieties**” There is no need to mention the site because it is available in your methodology part.

***Abstract***: is well written. However, you must consider the following specific points for its further improvement

1. Avoid the word “Results revealed that …” because the abstract by itself talks about the result. So it is redundancy.
2. Were these net benefits solely caused by the varietal effect? If so, why did the authors recommend using 100 kg of NPSB fertilizer alongside the variety? Please revise the points.
3. You carried out the experiment across two growing seasons, but you are still suggesting further research to create a strong recommendation. Please offer suggestions based on the findings you have at this time.

***Introduction:*** is well-written, and most of the in-text references used are recent and relevant. However, the authors should include some minor editorial comments on the attached manuscript. Additionally, they need to incorporate the most recent relevant findings to strengthen the study, highlight existing gaps, and clarify how their research addresses these gaps.

**Materials and Methods**

- Please edit the figures to improve their visibility for the readers.

Nodule numbers

- What led the authors to focus solely on the quantity of nodules as the nodulation parameters? Do you think that merely having an adequate number of nodules guarantees efficient nitrogen fixation?
- Please revise data collection methods for better clarity.
- The economic analysis need details by incorporating all important formulas.

**Results and discussion**

- Please verify that symbols and decimals are accurate throughout the manuscript and presented consistently.
- Results should be followed by tables, which must be positioned correctly.

**Conclusion**

- The writing is good; however, it should summarize the research instead of presenting the entire result. Additionally, please consider the feedback provided on the attached manuscript.

**References**

- Should be checked against the Journal guideline.
