# Supplementary material for: Optimizing NPSB fertilizer rates for enhanced yield and yield components of mung bean (Vigna radiata L:) varieties
Source: PLoS One. 2025 Oct 24;20(10):e0321243. doi: 10.1371/journal.pone.0321243 (PMC12551827; doi:10.1371/journal.pone.0321243)
Supplement: Attachment — Submitted filename: Responses for comments.docx [file pone.0321243.s003.docx]

**Responses are written in italics with blue color**

Manuscript ID: PONE-D-25-10453

Manuscript title: **Optimizing NPSB Fertilizer Application Rates for Enhanced Yield and Yield Components of Mung Bean (Vigna Radiata L.) Varieties in the Dalocha District of Central Ethiopia**

**General comments**

The manuscript is relevant and well-written, with very few corrections required.

**Specific comments**

**Title**

- The title should be smart and specific, so I suggest changing it to “**Optimizing NPSB Fertilizer Rates for Enhanced Yield and Yield Components of Mung Bean (*Vigna radiata* L.) Varieties**” There is no need to mention the site because it is available in your methodology part.

*We sincerely appreciate your constructive feedback regarding the title modifications. Your insights have been appreciated, and we have taken them into account as we revised my article. Thank you for your thoughtful contributions.*

***Abstract***: is well written. However, you must consider the following specific points for its further improvement.

1. Avoid the word “Results revealed that …” because the abstract by itself talks about the result. So it is redundancy. We did it
2. Were these net benefits solely caused by the varietal effect? If so, why did the authors recommend using 100 kg of NPSB fertilizer alongside the variety? Please revise the points. *We modified and accepted your comments.*
3. You carried out the experiment across two growing seasons, but you are still suggesting further research to create a strong recommendation. Please offer suggestions based on the findings you have at this time. *We accepted your comments, and thank you*

***Introduction:*** is well-written, and most of the in-text references used are recent and relevant. However, the authors should include some minor editorial comments on the attached manuscript. Additionally, they need to incorporate the most recent relevant findings to strengthen the study, highlight existing gaps, and clarify how their research addresses these gaps.

**Materials and Methods**

- Please edit the figures to improve their visibility. y for the readers. *We did it*

Nodule numbers

- What led the authors to focus solely on the quantity of nodules as the nodulation parameters? Do you think that merely having an adequate number of nodules guarantees efficient nitrogen fixation?
- Please revise data collection methods for better clarity.

*Your insights and considerations are greatly appreciated. We have gathered various nodulation parameters, including the number of nodules per plant, among other metrics. Our primary focus has been on the number of nodules, as this aligns closely with our findings regarding nodulation effectiveness and the role of leghemoglobin. This emphasis is crucial because it allows us to better understand the relationship between nodulation and plant health, ultimately contributing to our broader research objectives.*

- The economic analysis need details by incorporating all important formulas. *We did it. Thank you.*

**Results and discussion**

- Please verify that symbols and decimals are accurate throughout the manuscript and presented consistently. *We assessed it. thank you*
- Results should be followed by tables, which must be positioned correctly.

**Conclusion**

- The writing is good; however, it should summarize the research instead of presenting the entire result. Additionally, please consider the feedback provided on the attached manuscript. *Thank you.*

**References**

- Should be checked against the journal guideline. *Done, thank you*
